# Supplementary material for: Auditory Brainstem Responses (ABR) of Rats during Experimentally Induced Tinnitus: Literature Review
Source: Brain Sci. 2020 Nov 24;10(12):901. doi: 10.3390/brainsci10120901 (PMC7760291; doi:10.3390/brainsci10120901)
Supplement: Supplementary file 1 [file brainsci-10-00901-s001.pdf]

Supplementary Files

Table S1. Extracted data regarding tinnitus characteristics and tinnitus-relevant changes in ABR.

Article	Objective of the study	Tinnitus inducing	ABR aim (amplitude, latency, thresholds)	ABR time-point	Additional drugs (side effects based on the article)	Anesthesia during ABR *during noise exposure	Additional methods applied in the study	Experimental groups	Animals (age***, sex and strain)	Sample size: overall /used for ABR	Changes in threshold	Changes in amplitude	Changes in latency	Link between ABR and tinnitus recognizing	Tinnitus recognizing	Tinnitus time-point	Tinnitus-relevant results
Zhang et al. 2020 [36]	to study if loss of cochlear ribbon synapses contributes to tinnitus	SAL (200 mg/kg/d) for 10 consecutive days (intraperitoneal injection)	threshold, amplitude (wave I 90 dB for each frequency)	2 hours after the last SAL administration (on the 10th day)	-	Ketamine (90 mg/kg) + Xylazine (10 mg/kg)	ASR, IF of IHC	SAL (n=10), control (n=10)	8 weeks old male Wistar rats (250-300g)*	20/20	No significant differences between the SAL and control group	↓ wave I after SAL at all frequencies	No significant difference between SAL and control group	Data not available	GPIAS	after the last SAL administration	The mean ratio was significantly lower in the treated group than in the control group, 0:5145 +/- 0:045 and 0:68 +/- 0:0466, respectively.
Zhang et al. 2020 [54]	to examine if sodium salicylate disrupts expression of VGLUT3 and if it may corresponds	SAL (200 mg/kg/d) for 10 consecutive days (intraperitoneal	threshold	2 hours after the last SAL administration (on the 10th day)	-	Chloral hydrate (6 %, 0.6 ml/100 g)	WB, IF of cochlea	SAL (n=12), control (n=12)	8 weeks old male Wistar rats (250-300g)*	24/15	No significant differences between the SAL	Data not available	Data not available	Data not available	GPIAS	2 h after SAL administration on the 10th day	Mean inhibitory rates differed significantly among SAL

	to increased risk for tinnitus	injection)									and control group at 4, 16 and 20 kHz; ↓at 8, 12 (<10 dB) and 24 kHz (nearby 10 dB) in comparison to control group						(0.51 +/- 0.045) and control (0.68 +/- 0.047).
Castañeda et al. 2019 [37]	to study AEPs alterations, assessing early to late latency responses, in animals with tinnitus	SAL (350 mg/kg/d) for 3 consecutive days (orally by gavage)	threshold, amplitudes and latencies (I-V, 80 dB for each frequency)	Before, daily during SAL administration (1-3 days) and daily for 3 days during cessation of SAL (4-6 days)	-	Ketamine (88.88mg/kg) + Xylazine (10.36mg/kg)	MLR, LLR	SAL (n=27)	8 weeks old female Sprague-Dawley (SD) rats	27/15	↑ at all frequencies (1-3 days); on 6th day-no differences, except 16 kHz in comparison to pre-treatment	↓ wave I during SAL; ↑ wave II at frequencies >16 kHz only on 1st day; ↑ wave IV at 8-24 kHz on 1st day; wave V- no differences	← I-IV waves at 8, 16 and 24 kHz (1-4 days); → II-IV at 32 kHz (5-6 days)	There were correlations between tinnitus behavioral and ABR latencies (wave II at 8,16 kHz, III wave at 8 kHz, IV at 4,8 and 16 kHz) and amplitude (II and III at 8 kHz, IV at 16 kHz, V at 32 kHz). No	Pressing a lever in the presence of a tone stimulus (8 kHz) and to not press during	once a day for 7 days at the same time each day 3h after SAL administration	The false-positive response numbers significantly increased during SAL and cessation vs. control group till 6 day (no longer statistically significant in SAL vs. day 0).

														correlation between wave I and hearing threshold			
Duron et al. 2020 [44]	to test if together with lowering ASR threshold, salicylate injection would transiently affect brainstem responses in a consistent, hyperacusis-dependent manner	SAL (150 mg/kg/d) (intraperitoneal injection)	threshold, amplitude, latency (I- IV, 80-40 dB for each frequencies)	0.5 hour before, 0.5, 1 and 1.5 hours after SAL administration	-	Ketamine (10 mg/kg) + Xylazine (20 mg/kg)	MEMR, ASR	salicylate (n=19), control (n=18)	16 weeks old male Sprague-Dawley rats (250-300 g)**	37/15	30 min after SA:L no differences, 60 and 90 min after SAL: ↑ at 6 kHz (+8.5 dB); at 10 kHz (+10 dB); at 12 kHz (+17.5 dB); at 16 kHz (+18.3 dB)	90 min after SAL: ↑IV wave, ↓ I wave at 16 kHz	60 min after SAL: ← III-IV (0.25 ms) at 6 kHz; 90 min after SAL: ← IV at 10 kHz; 60 and 90 min after SAL→I at all frequencies (except 16 kHz)	Data not available	not applied	-	-
Lee et al. 2019 [46]	to examine the effects of EGb 761 (EGb) on the plasticity of NMDA receptor subunit 2B (GluN2B) in IC after SAL	SAL (350 mg/kg/d) (intraperitoneal injection)	threshold	5 days before and 2 hours after SAL administration	Egb 761 (a standard form of GBE) (40 mg/kg) for 5 consecutive days (orally) (data not available)	Zoletil (40 mg/kg) + Xylazine (10 mg/kg)	WB, IHC of IC	S (n=6), Egb (n=6) S+EgB (n=6), control (n=6)	7 weeks old male Sprague-Dawley (SD) rats	24/24	After SAL: ↑ +10 dB; no significant differences among groups but it is a	Data not available	Data not available	Data not available	not applied	-	-

	administration										tendency to higher threshold shift in SAL at 8 kHz than in control group						
Jang et al. 2019 [45]	to study the effects of memantine on salicylate-induced tinnitus model	SAL (400 mg/kg/d) for 7 consecutive days (intraperitoneal injection)	threshold	before, on 7 day (the last day of SAL) and on day 8 (the first day of cessation)	memantine (inhibitor of NMDA) (5mg/kg/d) for 7 consecutive days (injection) (data not available)	Data not available	rt-PCR, IHC of AC, NBPIAS	SAL+MEM (n=10), SAL (n=10)	9 weeks old male Sprague-Dawley (SD) rats (300 g)**	20/20	SAL: ↑ at 8 and 16 kHz (about + 10 dB) on 7 and 8 days (vs. before). SAL+MEM: no significant changes (vs. before)	Data not available	Data not available	Data not available	GPIAS	before and 2h after each SAL administation and 24h post-treatment	Baseline: 46% (control group), 47% (experimental group). After SAL: reducing, lesser extent in the group treated with a combination of SAL+MEM (at day 1,3,5 and 7 day).
Fang et al. 2016 [53]	to study tinnitus-like behavior in rats after salicylate treatment	SAL (300 mg/kg/d) for 4* or 8 consecutive days	amplitude (III wave)	before, after 8 days and 1 week after 8	not applied	Ketamine (80 mg/kg)+ Xylazine (4 mg/kg)	DPOAE, IHC of VCN, Transmission Electron	Repeated injections of SAL for 4 days (S4) (n = 10) or 8	6-8 weeks old male Wistar	42/6	Data not available	↓ at all frequencies after SAL, greater	Data not available	When GPIAS disappeared, amplitude recovered	GPIAS	4h after the final drug administration	Inhibition of GPIAS was shown in all

	and compare with DPOAE and ABR results	(intraperitoneal injection) *not ABR	days SAL administration		Microscopy (TEM), WB for VPC	days (n=10, recovery group with 1 week after 8 SAL days(n = 12), control (n=10)	(Wis) rats (200-280 g)*		at 8 and 16 kHz than at 24 and 32 kHz after 8 days. 1 week after treatment recovered to baseline levels	to baseline level	on 4 and 8 day	groups after SAL treatment. The S4 group showed a significant reduction in the mean percentage of the GPIAS vs. control group at 16kHz. The S8 group showed a reduction at 12 kHz and 16 kHz but not at 8 kHz or 20 kHz. The recovery group- no differences (It suggest that tinnitus-behavior

																	disappeared 1 week after SAL treatment).
Liu and Chen, 2015 [55]	to develop an objective method for tinnitus assessment using ABR and acoustic masking	SAL (300 mg/kg/d) (intraperitoneal injection)	latency and amplitude (I-V waves)	before and 2 hours after SAL administration	vigabatrin (a GABA transaminase inhibitor) disolved in drinking water for more than 2 weeks. For each rat 49+/-1.8 ml per day (orally) (data not available)	Chloral hydrate (400 mg/kg)	Diotic paradigm (forward masker and the probe were presented to both ears), dichotic paradigm (forward masker presented to one ear and the probe to the other ear)	Diotic paradigm (n=33), dichotic paradigm (n=10), standard ABR (n=11), diotic+vigabatrin (n=9)	2-5 months old female and male Wistar (Wis) rats	69/69	After SAL: ↑about 10-25 dB at all tested frequencies (no maskers)	Before SAL: ↓amplitude overall (diotic paradigm), no changes in dichotic paradigm; After SAL: stop decreasing,↑ at 16,24 and 32 kHz (diotic paradigm); ↓I wave at all frequencies (all paradi	Overall →; ←I-II and II-IV at some high frequencies (no maskers)	After SAL treatment GPIAS was reduced at high frequencies at which the forward masker enhanced the ABR in the diotic condition.	GPIAS	before and 2h after SAL injection	Reducing GPIAS at 16,24 and 32 kHz, but not at 6 and 12 kHz. Fully recovered 2 days after SAL treatment.

												gms); ↓ wave II at 16, 24 and 32 kHz (no masker); ↓ wave IV at 16 and 24 kHz (no masker)					
Ralli et al. 2014 [47]	to determine if memantine would affect salicylate-induced tinnitus; to determine what effects memantine or the combination of memantine and salicylate would have on DPOAE and ABR	SAL (300 mg/kg/d) for 4 consecutive days (injection)	threshold (to check long term changes after treatment)	before and 14 days after the last SAL administration	memantine (MEM) (NMDA channel blocker) (5 mg/kg/d) for 4 consecutive days (injection) (data not available)	Ketamine (10mg/kg)	DPOAE, NBPIAS	SAL (n=12), MEM (n=12), SAL+MEM (n=12)	3-5 months old male Sprague Dawley (SD) rats	36/36	No differences between pre- and on 15th days post treatment among the SAL, MEM and SAL+MEM groups (It indicates that SAL, MEM and SAL+M	Data not available	Data not available	Data not available	GPIAS	before, 2,24,48,72 and 96h after the first SAL administration and 24h after the last SAL injection	Before: 44, 42, 38, 39% for 6,12,16 and 20 kHz. After SAL significant reduction at 16 kHz. A significant reduction occurred between 2 and 72h with a peak at 48 hours only at 16 kHz;

											EM not produce long changes in hearing thresholds)						GPIAS returned near to baseline levels 24h after the last day of drug administration (35%). MEM: no significant changes in GPIAS vs. before. SAL+MEM showed less reduction in GPIAS vs. SAL (during the first 48h at 16 kHz).
Sawka and Wei, 2014[48]	to study whether post-salicylate changes observed in AC are also present in brainstem	SAL (250 mg/kg/d) (intraperitoneal injection)	amplitude (II and V wave)	before, 2 hours, 1 and 3 days after SAL administration	not applied	no narkose, restraint	AC recording	SAL (n=4)	6-7 months old male Sprague-Dawley	4/4	Data not available	↓wave V at 12 kHz (on the 1st and 3rd days the reducti	Data not available	no, study group consisted of 4 animals	not applied	-	-

									(SD) rats			on was greater than 2h); ↓wave II at 4,16 and 20 kHz (2 hours, 1 and 3 days after SAL)					
Liu and Chen, 2012[34]	to examine the effect of forward masking on the ABR in NaSal-induced tinnitus and in the presence of a continuous background tone	SAL (300 mg/kg/d) (injection)	amplitude, latency (I-V waves)	2 hours after SAL administration	not applied	Chloral hydrate (400 mg/kg)	not applied	Diotic paradigm (n=5), dichotic paradigm (n=6)	8-13 weeks old female and male Wistar (Wis) rats	11/11	Data not available	Before SAL: ↓ at all tested frequencies (diotic paradigm). After SAL: stop decreasing, ↑ at 16 kHz (masker: 16 kHz, 55 dB spl, 50 and 80 ms)	Before: overall → (diotic paradigm); after SAL: no changes (diotic paradigm)	Data not available	not applied	-	-
Chen et al. 2010 [43]	to study salicylate-induced tinnitus	SAL (200 mg/kg/d) for 5 days per	amplitude (III wave)	before, 3 days, 2 and 4 weeks	not applied	Isoflurane (4%)	CAP, DPOAE, hair cell	SAL 200 mg (n=15), other	3 months old male	23/6 (only young	Data not available	↓(4-32 kHz, 100 dB) on	Data not available	Data not available	not applied	-	-

		week for 3 weeks; 200,250,300 or 350 mg/kg/d for 2 or 3 weeks* (injection) *no ABR in those groups)		after SAL administration			assessment	doses (n=8)	Sprague-Dawley; 3 and 18 months Fischer 344/NHsd (F344) rats	SD rats)		3 days, 2 and 4 weeks after SAL, the greatest reduction at 4 and 8 kHz. Not recovery between 3 days and 4 weeks post-salicylate					
Ralli et al. 2010 [31]	to compare salicylate and quinine induced tinnitus in rats	SAL (300 mg/kg/d) for 4 consecutive days (injection)	threshold (to confirm that the drug treatments did not cause any permanent effects)	before and 2 weeks after last SAL administration	quinine (200 mg/kg/d) for 4 consecutive days (orally) (data not available)	Isoflurane (1.5%)	DPOAE, NBPIAS	SAL (n=12)	3-5 months old male Sprague Dawley (SD) rats	24/24	No significant changes	Data not available	Data not available	Data not available	GPIAS	before, 2,24,48,72 and 96h after the first SAL administration and 24h after the last SAL injection	Baseline: 36,33% at all tested frequencies (SAL). After SAL: tinnitus with a pitch near 16 kHz starting 2h posttreatment,

																	persisting over the 4-day treatment period and disappearing 24h after the last injection (30%).
Bauer et al. 2000 [61]	to study the GAD and GABAA receptor in auditory brainstem structures of rats chronically treated with SAL	SAL (8mg/ml) in drinking water for 4 weeks or 4 months* (orally) *no ABR)	Threshold	1 week before being sacrified (details not available)	not applied	Ketamine (130 mg/kg) + Xylazine (26 mg/kg)	WB, GABAA receptor autoradiography, SAL level in serum	SAL (n=15), control (n=15)	7-12 weeks old male Long Evans (LE) rats	30/10	After SAL: ↑ at 4-31,5 kHz vs. control group (mean: 38.8 dB vs. 35 dB)	Data not available	Data not available	Data not available	not applied	-	-
Kim et al. 2020 [50]	to identify acute tinnitus and evaluate the efficacy of steroids for noise-induced acute tinnitus	narrow-band noise: 16 kHz, 112 dB SPL, 4h, speaker (bilateral)	threshold (to determine if noise trauma could induce auditory threshold shift)	before, 1 day after noise exposure, 1 and 10 days after completing DEX administration	1.5 mg of DEX/kg daily for 5 days after noise exposure (intraperitoneal) (data not available)	Zoletil 50 (0.1 cc/100 g) +Rompun 2% (0.02 cc/100 g)*	IHC of cochlea (additional 8 weeks-old Wistar rats as a control)	Noise (n=7), noise+DEX (n=12)	8 weeks old male Sprague-Dawley (SD) rats	19/19	Before noise: at 8,16 and 32 kHz (15 dB); 1 day after noise: no differences vs. before. On 1 and 10 days after	Data not available	Data not available	Data not available	GPIAS	before, 1 day after noise and 1 and 10 days after completing DEX administration	Before noise: 51 +/-13%; before noise+DEX: 47 +/-14%.At 1 day after noise: no differences between groups. At 10 days: the noise +

											DEX no differences between groups except 32 kHz at 1 day after (DEX+ noise: about 20dB, noise: <10 dB)						DEX group (36 +/- 14%) was significantly greater than that in the noise (16% +/- 19%).
Brozoski et al. 2019 [62]	to examine the link between tinnitus and auditory vigilant attention as well as auditory selective attention	narrow-band noise: 16 kHz, 120 dB SPL, 1h, unilateral (contralateral ear-plugged)	threshold	before, immediately and 14 months after noise exposure	not applied	Isoflurane/O_2 (1.7%) mixture *	attention task	Noise (n=8), control (n=8)	3 months old male Long Evans (LE) rats	16/16	Immediately after noise in both ears ↑; 14 months after in ipsi-ears ↑, contr-ear no	Data not available	Data not available	There were not differences in threshold between tinnitus (+) and no-tinnitus(-) rats after noise.	an operant conditioned-suppression procedure	Before, 3 and 9 months after noise	Tinnitus animals showed a significant downshift vs. control and no-tinnitus rats before, and 9 months after. The exposed no-Tinnitus animals were not significantly

																	different from the unexposed controls.
van Zwieten et al. 2019 [52]	to study the effect of high frequency stimulation (HFS) and low frequency stimulation (LFS) of the medial geniculate bodies (MGB) on noise-induced tinnitus	narrow-band noise: 16 kHz,115 dB, 1.5 h, unilateral (contralateral ear-plugged)	threshold (to estimate hearing function)	before and 6 weeks after noise exposure	not applied	Ketamine (90 mg/kg) + Xylazine (10 mg/kg) *	Elevated Zero Maze (EZM), Open Field (OF)	Noise (n=11)	11 weeks old male Sprague-Dawley (SD) rats (350 g)**	11/11	After noise in the ipsilateral ear ↑ at all frequencies (threshold: 70-90 dB); no differences between before and and contr-ears (threshold: 40-50 dB)	Data not available	Data not available	Data not available	GPIAS	before (with HFS and without) and 4-6 weeks after noise during HFS, post-HFS and post-LFS	All rats showed tinnitus. Only at 16kHz (background noise) were significantly changes. (increasing after noise exposure). At baseline, there was no effect of HFS.
van Zwieten et al. 2019 [51]	to investigate the effect of HFS of the DCN on a noise-induced tinnitus	narrow-band noise: 16 kHz,115 dB, 1.5 h, unilateral (contralateral ear-plugged)	threshold	before and 2 weeks after noise exposure	not applied	Ketamine (90 mg/kg) + Xylazine (10 mg/kg) *	IHC of IC, AC, MGB (c-Fos)	Noise +HFS (n=6), noise (n=5)	11 weeks old male Sprague-Dawley (SD) rats (350 g)**	10/10	Before noise: 40-60 dB; after noise: ↑ in ipsi-ears (70-100 dB); contr-ear and	Data not available	Data not available	Data not available	GPIAS	before (with HFS and without) and 4-6 weeks after noise during HFS, post-HFS	GPIAS were significantly increased after noise exposure (only at 16 kHz as a backgro

										before noise (40-50 dB)					und). After noise during HFS: the gap:no-gap ratio decreased (16 kHz as a background). At baseline, there was no effect of HFS.
Ahsan et al. 2018 [49]	to demonstrate therapeutic effects of deep brain stimulation (DBS) on noise-induced tinnitus	band noise: 8-16 kHz, 115 dB, 2 h, unilateral (contralateral ear-plugged)	threshold	before, immediately and 1 week after noise exposure	not applied	Isoflurane (2%–3%) *	not applied	Noise (n=6), control (n=3)	11-15 weeks old male Sprague-Dawley (SD) rats (350–450g)**	9/6	Before and after in contr-ears (threshold: 20-40 dB), immediately after noise in ipsi-ears ↑ (threshold: 50-60 dB); recovered 1 month later	Data not available	Data not available	Data not available	GAP	after noise exposition	4/6 rats showed tinnitus. There was a significant difference at 12 kHz, 20 kHz, 28 kHz, and at broadband noise (BBN).
Turner and Larse	to explore the relative impacts of	1st experiment:	Threshold	before and immedi	not applied	Isoflurane (poor results)	not applied	1 part: Noise (n=137),	5-6-months old	1st experiment:	Only noise 122 dB	Data not	Data not available	Data not available	GPIAS	on 1, 3, 7, 14, 21, 28 after	The 12 noise exposure

n, 2016 [65]	noise exposure duration, intensity, and spectrum on developing tinnitus and hyperacusis 12 months later in middle-aged rats	narrow-band noise: 16 kHz, 110, 116 or 122 dB SPL for 0.5h, 1h or 2 h, unilateral (contralateral ear-not announced); 2nd experiment: 8 or 32 kHz or BBN at 110 dB for 0.5h		ately after noise exposure		then Ketamine + Xylazine (doses-not available) *		control (n=30); 2 part: noise (n=48), control (n=18)	male Fischer 344/Brown Norway F1 rats	137/137; 2nd experiment: 66/66	for 2h evoked significantV↑ at 32 kHz	available				noise exposure and monthly thereafter over the course of 1 y until they reached middle age at 18 months old (both part of experiment)	conditions resulted in tinnitus prevalence rates 12-mo later ranging from a level similar to control controls at 11%, and up to 50%. Hyperacusis rates ranged from 7 to 33% (no significant differences vs. control group).
Bing et al. 2015 [33]	to understand the therapeutic potential of NMDA receptor inhibition in tinnitus therapy using	narrow-band noise: 10 kHz, 120 dB, 2 h, unilateral (contralateral ear-plugged)	threshold, amplitude (I-IV waves); corF to estimate the recove	6 days before and 15 days after noise exposure	1 or 2 times of 200 µM AM-101 (NMDA receptor antagonist) 2,4 and 8 days after noise exposure	Ketamine hydrochloride (75 mg/kg) +Xylazine hydrochloride (5 mg/kg) *Medeto	IHC of cochlea, counting ribbon synapses	8 different experimintal groups	8-12 weeks old female Wistar (Wis) rats (200-300g)*	42/42	After noise ↑ at 8 kHz and higher frequencies. No difference	After noise: ↓wave I, more pronounced in rats without AM-101; ↓IV	Data not available	Animals with reduced tinnitus showed a less severe reduction of the numbers of IHC	the motor task	Calculating the delta of the tinnitus behavior as relative activity during silence 3	A little improvement in tinnitus behavior.

	tinnitus in a noise trauma model		ry of the ABR waves		(applied locally to the round-window) *interference between AM-101 and ketamine	midine hydrochloride (0.33 mg/kg)					between rats treated with AM-101 and not-treated	wave. In rats treated with AM-101 48h after noise ABR was partially conserved		synaptic contacts		and 10 days after exposure	
Zheng et al. 2015 [59]	to test if a combination of delta-9-tetrahydrocannabinol (delta-9-THC) and cannabidiol (CBD), delivered in a 1:1 ratio, could affect acoustic trauma-induced tinnitus in rat model	narrow-band noise: 16 kHz, 115 dB, 1 h, unilateral (contralateral ear-plugged)	threshold	before, immediately and 6 months after noise exposure	delta-9-THC (1.5 mg/kg) and CBD (1.5 mg/kg) every day for a total of 27 days (no ABR in drugs' groups)	*Fentanyl (0.2 mg/kg) + Medetomidine hydrochloride (0.5 mg/kg)	not applied	Noise (n=30), control (n=20)	7-10 weeks old male Wistar (Wis) rats (300–350 g)*	50/50	Immediately after noise:↑ ipsi-ear (threshold: 50-70 dB). Contr-ear: no changes (threshold: 20-30 dB). 6 months after noise: recovery of the ABR	Data not available	Data not available	Data not available	a conditioned lick suppression task	1 month after noise trauma	14 rats presented tinnitus. The cannabinoids significantly increased the number of tinnitus animals in the exposed-tinnitus group, but not in the sham group.
Zheng, McPh	to study the effect of L-baclofen	narrow-band noise: 16	threshold	before, immediately	L-baclofen (5 mg/kg/d)	Ketamine hydrochl	IHC of cochlear	Noise (n=8), noise+L-	8-10 weeks old	32/missing data	Immediately after	Data not	Data not available	Data not available	a conditioned	2 weeks and then at	2 and 17.5 weeks

erson and Smith, 2014 [58]	adminitration at early time points on developing tinnitus after noise trauma	kHz, 115 dB, 1 h, unilateral (contralateral ear-plugged)		and 22 weeks after noise exposure	0.5h after the noise and then again every 24 h for 5 days and at 17.5 weeks following the noise or sham exposure for 4.5 weeks (3mg/kg/d) *sedation in response to the 5-mg/kg dose (pilot study)	oride (75 mg/kg) + Medetomidine hydrochloride (0.3 mg/kg) *Medetomidine hydrochloride (0.33 mg/kg)	nucleus (CN)	Baclofen (n=8), control (n=8), L-Baclofen + sham (n=8)	male Wistar (Wis) rats (300-350 g)*		noise: ipsi-ears (threshold 50-70 dB), contr-ears: no changes; 22 weeks after in both ears restored to baseline levels (temporary elevation)	available			lick suppression task	10 and 17.5 weeks after noise exposure	after noise exposure: shift of the lick suppression (in resposne to 20-kHz, but not BBN or 32 kHz). Tested again at 10 weeks following the acoustic trauma, all three stimuli resulted in greater lick suppression in exposed animals (it indicated the presence of tinnitus at multiple frequencies).

Laundrie and Sun, 2014 [38]	to investigate the link between the onset of the changes in central auditory system and the onset of the tinnitus	narrow-band noise: 12 kHz, 120 dB SPL, 1h, unilateral (contralateral ear-plugged)	threshold (to assess the degree of the noise-induced hearing loss)	5-6 hours after noise exposition and daily until rat had recovered to its full ability	not applied	Isoflurane (1-2%)*	AC recording	Noise (n=3)	3-4 months old male Harlan Sprague-Dawley (HSD) rats	3/3	Before noise: 20 dB; 2h after noise: ipsi-ears ↑at 12 kHz (+ 30-45 dB), contr-ears (+ <10 dB) at 12 kHz . All rats developed either permanent (>2 weeks) or temporary (<3 days) hearing loss in the exposed ear(s)	Data not available	Data not available	Data not available	GPIAS	before and 4h after noise exposure	4h after noise: GPIAS decreased to 31% at 6 kHz, 28% at 12 kHz, 36% at 16 kHz*, and 42% at 20 kHz*. Reduction remained constant (~ 15% decrease compared to pre-exposure) . *significant
Ropp et al. 2014 [39]	to study the long-term effects of sound-induced cochlear trauma on spontaneous discharge	narrow-band noise: 16 kHz, 116 dB SPL, 2h, unilateral (contrala	threshold	before and 1 week after noise exposure	not applied	Ketamine (40 mg/kg)+ Xylazine (10 mg/kg) *unanesthetized (rat was	IC recording	Noise, control (not clearly announced)	adult male Sprague-Dawley (SD) rats	not clearly announced	After noise: ↑ at 10-40 kHz (variable patterns of changi	Data not available	Data not available	Data not available	GPIAS	before and at various delays after sound exposure (2-3 times	GPIAS scores returned to normal baselines in the weeks immedia

	rates in the central nucleus of the inferior colliculus (IC)	teral ear-plugged)				held in a slowly rotating hardware cloth cage)					ng); contr-ears- no changes					per week for 1-4 months)	tely following sound exposure. Unilateral threshold shifts did not strongly influence GPIAS. The GPIAS profile did not deviate from pre-exposure baselines when the rat was tested 4 and 9 weeks after sound exposure.
Ruttiger et al. 2013 [60]	to identify a tinnitus-specific difference between equally acoustically exposed animals with and	narrow-band noise: 10 kHz, 120 dB SPL, 1h or 1.5 h, binaural	amplitude (click, 90 dB SPL), threshold (corF)	6 days after noise exposure (group 1h), 30 days after noise exposur	not applied	Ketamine (75 mg/kg) + Xylazine hydrochloride (5 mg/kg)*	IHC of AC and cochlea (Arc, synapses), ribbons counts	noise 1h (data not available) , 1.5h noise (data not available)	8-12 weeks old female Wistar (Wis) rats	32/32	After noise: ↑in all groups. The group of tinnitus rats expose	↓ overall (corF); a reduced recovery for tinnitus	Data not available	IHCs ribbon loss (deafferentation) did not lead to tinnitus when ABRs were	the motor task	before and at 6 day (1 h) or 30 days (1.5 h) after exposure	5 of 15 rats and 5 of 17 rats for the 1 h and 1.5 h exposure had developed a

without tinnitus using ABR	e (group 1.5 h)		(200-300g)*	d to 1h had a significantly larger hearing loss than no-tinnitus rats. Hearing loss for frequencies above 11.3 kHz was significantly increased compared to no-tinnitus groups. After the more intense noise exposure (1.5 h), threshold loss in tinnitus	animals in comparison to tinnitus-free animals was observed.	restored and Arc was mobilized in the AC. When brainstem responses remain reduced and Arc was not mobilized, IHC ribbon loss resulted in tinnitus	significantly elevated silence activity, indicating tinnitus.

											animals was also significantly greater for low stimulus frequencies						
Pace and Zhang, 2013 [64]	to study the effect of intense tone-induced tinnitus, hearing loss, and hyperacusis-like behavior on spatial learning and memory, and anxiety	narrow-band noise: 10 kHz, 118–120 dB peSPL, 2 h; 5 weeks later the 2nd exposure for 3 h unilateral (contralateral ear-plugged)	threshold	1 and 8 weeks after 2nd noise exposure	not applied	Isoflurane (5%)* 2nd noise: during awake	elevated plus maze (EPM), Morris Water Maze (MWM)	Noise (n=8), control (n=7), excluded (n=4)	2-2.5 months old male Long-Evans (LE) rats	29/25	After noise: click-no changes; 1 and 8 weeks after: ↑ at 8-28 kHz(rats with tinnitus) 12-28 kHz (rats without tinnitus) in ipsi-ears. Click-no changes. Rats with tinnitus presented	Data not available	Data not available	No special link between ABR in tinnitus groups and GPiAS results.	GPIAS	before and at 1 day after and 2 times a week till 6 weeks post-the second exposure	12/18 rats presented tinnitus (post-exposure GPIAS ratios that were both significantly higher than pre-exposure GPIAS ratios and were not significantly lower than post-exposure startle only ratios).

											higher threshold than rats without tinnitus (8 weeks after) . Contr-ears (no changes vs. before noise)					
Singer et al. 2013 [71]	to study the impact of different degrees of cochlear damage and the influence of stress on tinnitus induction	narrow-band noise: 10 kHz, 80,100, 110, or 120 dB SPL for 1-2 h, binaural	corF to estimate the recovery of the ABR waves	before and 6-14 days after noise exposure	not applied	Ketamine hydrochloride (75 mg/kg) + Xylazine hydrohloride (5 mg/kg)*	Northern Blot, urine analysis, IHC of cochlea and AC, ribbon counts, social stress	noise (n=68-71), control (n=18-21)	8-12 weeks old female Wistar (Wis) rats (200-300 g)*	89/34	6-14 days after noise (120 dB): ↑ (threshold: 31-56 dB) vs. Control group (threshold: 5-14 dB)	6-14 days after noise (110 dB SPL)- the reduced ABR waves was restored; noise (120 dB SPL) waveforms were significantly distorted and reduced in their amplitudes	IHC ribbon loss (deafferentation) leads to tinnitus when ABR functions remain reduced and Arc is not mobilized in the hippocampal CA1 and AC. If ABR waves are functionally restored	the motor task	6–14 days after noise exposure	Tinnitus occurred only in animals exposed at 120 dB SPL (vs. control group).

														and Arc is mobilized, tinnitus does not occur.			
Brozoski et al. 2012 [63]	to test the hypothesis that the DCN serves as a necessary trigger zone for the pathological cascade leading to tinnitus	narrow-band noise: 16 kHz, 116 dB, 1 h, unilateral (contralateral ear-plugged)	threshold	before and immediately after noise exposure	not applied	Data not available *Isoflurane (dose-data not available)	histology of DCN	noise (n=23), control (n=23), excluded (n=2)	3 months old male long-Evans (LE) rats	48/46	Immediately after noise: ↑ in ipsi-ears (+ 30 to 50 dB); contr-ears no changes. No significant differences between animals with and without DCN lesions	Data not available	Data not available	Data not available	an operant conditioned-suppression	immediately after the acoustic trauma	Significant evidence of tinnitus appeared at 20 kHz in the exposed DCN-intact animals. The exposed animals showed a frequency-specific downshift in their discrimination functions, compared to unexposed controls.
Zheng et al. 2012 [70]	to investigate the effects of memantine	narrow-band noise: 16 kHz, 110	threshold	before, immediately and 3	memantine: 5 mg/kg (injection) (no ABR in	Ketamine hydrochloride (75	not applied	Noise (n=8), control (n=8)	8-10 weeks old male	16/ data not	immediately after noise: ↑	Data not available	Data not available	Data not available	a conditioned lick	after the noise trauma	5/8 rats after noise exhibited

	on the behavioral manifestations of tinnitus induced by acoustic trauma	dB, 1 h, unilateral (contralateral ear-plugged)		months after noise exposure	drug groups)	mg/kg) + Medetomidine hydrochloride (0.3 mg/kg)*			Wistar (Wis) rats (300-350 g)*	available	at 16 and 20 kHz, contr-ears no changes. 3 months after: no differences vs. Control group				suppression task		tinnitus but acoustic trauma did not produce significantly more lick suppression than the control animals to the 20 kHz tones. A 5-mg/kg dose of memantine significantly reduced the proportion of these animals which exhibited tinnitus-like behavior (2/5 compared to 5/5).
Zheng et al.	to investigate the potential	narrow-band noise: 16	threshold	before and immedi	L-baclofen (1,3 or 5 mg/kg) 1 h	Ketamine hydrochl	not applied	Noise (n=8),	8-10 weeks old	16/16	After noise: ipsi-	Data not	Data not available	Data not available	a conditioned	before and 2 weeks	A significant

2012 [57]	of L-baclofen to treat tinnitus using an acoustic trauma animal model	kHz, 110 dB, 1 h unilateral (contralateral ear plugged)		ately after noise exposure	before tinnitus test injection (no ABR in drugs' groups) (data not available)	oride (75 mg/kg) + Medetomidine hydrochloride (0.3 mg/kg)*		control (n=8)	male Wistar (Wis) rats (300-350 g)*		ears ↑across all of the frequencies; contr-ears: no changes	available			lick suppression task	after noise exposition	downward shift in the supression ratio in tinnitus rats vs. control (in response to the 20 kHz tones but not to the BBN or 10 kHz).
Zheng et al. 2011 [56]	to study the effect of noise-induced tinnitus on spatial performance	narrow-band noise: 16 kHz, 110 dB, 1 h unilateral (contralateral ear plugged)	threshold	before and immediately after noise exposure	not applied	Ketamine hydrochloride (75 mg/kg) + Medetomidine hydrochloride (0.3 mg/kg)*	T-maze task test	Noise (n=8), control (n=8)	8 weeks old male Wistar (Wis) rats (250-300 g)*	16/16	↑in the exposed ears across all of the frequencies, no changes in contr-ears	Data not available	Data not available	Data not available	a conditioned lick suppression task	2 weeks and 10 months after the acoustic trauma	2 weeks and 10 months after noise there was a significant downward shift during 20 kHz tone but not for 10 kHz and BBN presentation.
Wang et al. 2009 [66]	to study the GlyR and gephyrin message	narrow-band noise: 17 kHz, 116	threshold	before, immediately and 16	not applied	Ketamine hydrochloride (50	IHC of DCN	Noise (n=14), control (n=15)	3-4 months old male	42/42	immediately after noise:	Data not available	Data not available	Data not available	GAP	20 days after sound exposur	10/14 sound-exposed rats

	and protein levels 16 weeks following a 1-hour sound-exposure in adult rats	dB SPL, 1h, unilateral (contralateral ear plugged)		weeks after noise exposure		mg/kg) + Xylazine (9 mg/kg)*			Fischer Brown Norway (FBN) rats		↑in exposed ears, recovered to baseline level at 16 weeks after noise exposure; contr-ears (no changes vs. before)					e every 2 weeks up to 16 weeks	showed significantly worse gap detection at 24 and 32 kHz 16 weeks following sound exposure.
Ouyang 2017 [42]	to study the mechanism of blast-induced tinnitus	blast exposure (194 dB SPL), unilateral (contralateral ear plugged)	threshold, amplitude (I wave at 28 kHz, 50-80 dB SPL)	before and 5 weeks after blast exposure	not applied	Isoflurane (5%) *Isoflurane (4%) or Ketamine (100 mg/kg)+ Xylazine (10 mg/kg)	Elevated Plus Maze (EPM), Morris Water Maze (MWM), Manganese-enhanced MRI (MEMRI)	Blast (n=13), control (n=6), excluded (n=11)	60-70-days old male Sprague-Dawley (SD) rats	30/30	5 weeks after blast-exposure no differences vs. pre-exposure (recovery). Anesthesia did not differ the results.	↓ wave I in ipsi-ear after blast-exposure (across most intensities) in tinnitus(+) and tinnitus(−) rats compared to controls.	Data not available	Data not available	GPIAS	before (3 times/week) and after (2 times/week) blast exposure	8/13 showed tinnitus. Gap-detection deficits occurred through five weeks post-blast at a frequency range of 10–28 kHz, although the 26–28 kHz region was the most

												Ketamine and isoflurane did not differ the results					common and robust frequency band among individual animals.
Mahmood et al. 2014 [40]	to investigate the therapeutic effects of sildenafil on noise-induced hearing loss	3 consecutive blast exposure unilateral (kHz, dB-data not available) (contralateral ear plugged)	threshold (o determine if sildenafil had any therapeutic effects on blast-induced threshold shifts.)	before, immediately, 1,3 and 6 weeks after blast exposure	Sildenafil (a phosphodiesterase-5 inhibitor): for 7 days after blast exposure (10 mg/kg/day) (orally) After the 7-day, 1 week without treatment then a second 7-day round during the third week post-blast (data not available)	Isoflurane (2–3%)*	-	Blast (n=6), blast+sildenafil (n=10), sildenapil (n=8), excluded (n=6)	110 days old male Sprague Dawley rats (250–300 g)	30/24	After blast: ↑ in both ears at all frequencies (all groups);1 week post-blast: still ↑ in the ipsi-ears (8-28 kHz) and in contr-ears (20-28 kHz) (all groups); 3 weeks post (blast+ sildenafil) ↑ at 8 and	Data not available	Data not available	Data not available	GPIAS	1 h after the last blast exposure and for 8 weeks afterward	At 1 week post-blast, both (blast and blast+sildenafil) showed changes in GPIAS at all frequencies vs. control. Rats (sildenafil+ blast) showed worse GAP impairment at 18–20 kHz while the rats (only blast) at 26– 28 kHz and

											16-28 kHz (ipsi-ears), untreated rats: ↑ at 16-28 kHz (ipsi-ears). From 3 to 6 weeks threshold shifts remained stable. At 6 weeks post blast+ sildenafil ↑ from 8 to 28 kHz, in blast group: ↑ from 16 to 28 kHz						worse PPI ratios at 6–12, 18–20, and BBN compared to rats (sildenafil+ blast). It implies stronger tinnitus at those frequencies for those groups, respectively. Worse PPI performance at several frequencies in the rats (only blast) indicated greater overall hearing impairment post-blast.
Mao et al.	to investigate the	a single blast exposure	threshold	before and 1,14,28	not applied	Ketamine (100 mg/kg)+	MRI	Blast (n=7),	60-70-days old	10/7	Before blast: mean	Data not	Data not available	Data not available	GPIAS	before and 1, 14, 28,	1 day after blast:

2012 [41]	underlying mechanisms of blast-induced tinnitus, hearing loss, and associated traumatic brain injury (TBI)	(10 msec, 194 dB SPL) bilateral	and 90 days after blast exposure	Xylazine (10 mg/kg)*	died (n=3)	male Sprague-Dawley (SD) rats	38.57 dB; 1 day after blast exposure: ↑(mean threshold: 61.57 dB); on 14, 28 and 90 days after blast recovered to 37.86 dB SPL, 30.71 dB SPL, and 27.86 dB SPL	available	and 90 days after blast exposure	GPIAS values significantly higher. GPIAS values showed a trend of increase at 22–24 kHz. Blast exposure also caused significant impairment in PPI. On post-blast day 14, significant GPIAS impairments only occurred at 28–30 kHz and BBN (higher-frequency regions) . PPI impairments

were maintained at 18–20 kHz , 22–24 kHz , and BBN . On post-blast days 28 and 90, GPIAS recover to pre-blast baseline levels at all the frequency bands tested. PPI was not impaired at 28–30 kHz.

Abbreviations: ↓, reduced; ↑, increased; -, no changes; →, prolonged; ←,reduced; VGLUT3, vesicular glutamate transporter 3; GAD, glutamic acid decarboxylase; HFS, high-frequency stimulation; LFS, low frequency stimulation; AC, auditory cortex; IC, Inferior Colliculus; NMDA, The N-methyl-D-aspartate receptor; WB,, Western Blot; DCN, Dorsal Cochlea Nuclei; IF, Immunofluorescence; HL, hearing loss; SAL, sodium salicylate; AEP, auditory evoked potentials; GPIAS, Gap pre-pulse inhibition of the acoustic startle reflex; IHC, immunohistochemistry; DEX, dexamethasone, MEM, memantine; NBPIAS, Noise burst prepulse inhibition of the acoustic startle reflex; COX-2, Cyclooxygenase-2; S4, 4 days; S8, eight days; MEMR, Middle ear muscle reflex; ASR, acoustic startle response; EPM, elevated plus maze; MWM, Morris Water Maze; TEM, Transmission Electron Microscopy; VCN, Ventral Cochlea Nuclei; Wis, Wistar rats; SD, Sprague-Dawley rats; LE, Long-Evans; ipsi, ipsilateral; contr, contralateral; MEMRI, Manganese-enhanced MRI; *Age was calculated based on the animals weight (https://www.criver.com/products-services/find-model/wistar-igs-rat?region=23); **Age was calculated based on. the animals weight (https://www.taconic.com/rat-model/sprague-dawley); *** age at the beginning of experiments.

Table S2. Extracted data regarding ABR.

Article	ABR System	Speaker (plugged into the ear canal or open field)	Type/ Duration	Rate	Intensity (decrements)	Frequency	Polarity	Repetitions per recording	Filters (notch filter)	Electrodes placement	Additional information
Zhang et al. 2020 [36]	TDT	Data not available	Data not available	Data not available	Data not available	2, 4, 8, 12, 16, 20, and 24 kHz	Data not available	Data not available	Data not available	3: vertex (active), ipsi and contr-pinnas (reference and ground)	Data not available
Zhang et al. 2020 [54]	TDT	directly to ear canal	tone burst/ 5 ms (2-1-2)	21/s	90-20 dB SPL (in 5 dB)	2, 4, 8, 12, 16, 20, and 24 kHz	Data not available	Data not available	Data not available	3: vertex (active), ipsi and contr-pinnas (reference and ground)	Data not available
Castañeda et al. 2019 [37]	IHS	directly to ear canal	tone burst/ 5 ms (2-1-2)	21/s	80-0 dB SPL (in 10 dB)	4, 8, 16, 24, and 32 kHz	Data not available	1024	HP-30 Hz, LP-3000 Hz (data not available)	4: vertex (active), pinna (references), back (ground)	maintaining body temperature at 37.5 ± 1 °C (non electrical heating pad), electrode impedance ranged between 1-3 kΩ
Duron et al. 2020 [44]	TDT	directly to ear canal	tone burst/ 6 ms (1-4-1)	17/s	80-0 dB SPL (in 10 dB)	6, 10, 12 and 16 kHz	Data not available	200	Data not available	3: vertex (active), mastoid (reference), back (ground)	Data not available
Lee et al. 2019 [46]	IHS	Data not available	tone burst/ data not available	21/s	90-10 dB SPL (in 5 dB)	8, 16, and 32 kHz	Data not available	512	HP-100 Hz, LP-1500 Hz (data not available)	3: vertex (active), ipsi and contra-ears (reference and ground)	The resistance between each electrode and the ground electrode <2 kΩ
Jang et al. 2019 [45]	TDT	directly to ear canal	tone burst/ 5 ms (2-1-2)	50/s	90-0 dB SPL (in 10 dB)	8 and 16 kHz	Data not available	512	HP-100 Hz, LP-3000 Hz (data not available)	2: vertex (active), occiput (reference)	Data not available
Fang et al. 2016 [53]	TDT	directly to ear	tone burst/ 5 ms (1-3-1)	21/s	70 dB SPL	8,16,24 and 32 kHz	Data not available	500	Data not available	3: the mastoid (active), apex nasi (reference), vertex (ground)	temperature was maintained at 25 °C in a sound-proof room
Liu and Chen, 2015 [55]	TDT	directly to ear	tone burst/ 5 ms (2-1-2)	Data not available	70 dB SPL	6, 12, 16, 24 and 32 kHz	Data not available	200	HP-300 Hz, LP-3000 Hz (50 Hz)	4: vertex (active), mastoids (references),	The resistance between each electrode and the

										nose tip (ground)	ground electrode <1 kΩ
Ralli et al. 2014 [47]	TDT	an open field: 1 cm from ear	tone burst/ data not available	10/s	100-0 dB SPL (in 10 dB)	6,12,16,24 and 32 kHz	Data not available	>1000	HP-100 Hz, LP-3000 Hz (data not available)	3: vertex (active), ipsi and contr-pinnas (reference and ground)	Data not available
Sawka and Wei, 2014 [48]	TDT	close to ear	tone burst/ 4 ms (data not available)	19/s	90 dB	4,8,12,16 and 20 kHz	alternating	512	HP-100 Hz, LP-3000 Hz (60 Hz)	atypical, AC electrodes were used	Data not available
Liu and Chen, 2012 [34]	TDT	open field: 10 cm from ears	tone burst/ 5 ms (2-1-2)	Data not available	50 dB SPL (masker) 70 dB SPL	6, 12 and 16 kHz	Data not available	200	HP-300 Hz, LP-3000 Hz (50 Hz)	3: vertex (active), mastoid (reference), nose tip (ground)	The resistance between each electrode and the ground electrode <1 kΩ
Chen et al. 2010 [43]	TDT	directly to ear	tone burst/ 5 ms (1-3-1)	21/s	100 dB SPL; 100-20 dB SPL (only at 12 kHz)	4, 8, 12, 16, 20 and 32 kHz	alternating	600	HP-10 Hz, LP-3000 Hz (60 Hz)	3: vertex (active), mastoid (reference), behind the shoulder (ground)	maintaining body temperature at 37°C (warm blanket)
Ralli et al. 2010 [31]	TDT	open field (data not available)	tone burst/ 5 ms (2-1-2)	21/s	100-0 dB SPL (in 10 dB)	6, 12, 16, 24 and 32 kHz	Data not available	1000	HP-100 Hz, LP-3000 Hz (data not available)	3: vertex (reference), ipsi and contr-pinnas (active, ground)	Data not available
Bauer et al. 2000 [61]	TDT	Data not available	click and tone burst/3 ms (1-1-1)	10/s	Data not available	4, 10, 15, 20 and 31.5 kHz	alternating	512	HP-300 Hz, LP-10000 Hz (Data not available)	3: vertex (active), nose (reference), neck (ground)	Data not available
Kim et al. 2020 [50]	TDT	Data not available	tone burst/ 5 ms (1-3-1)	11.1/s	(in 5 dB)	8,16 and 32 kHz	Data not available	512	Data not available	3: vertex (active), mastoid (reference), neck (ground)	Data not available
Brozoski et al. 2019 [62]	TDT and IHS	directly to ear canal	tone burst/ 5 ms (2.5-0-2.5)	20/s	95-5 db SPL (in 10 dB)	8, 10, 12, 16, 20, 24 and 32 kHz	Data not available	256	Data not available	3: vertex (active), bulla (reference), hind leg (ground)	Data not available

van Zwieten et al. 2019 [52]	Data not available	directly to ear canal	tone burst/ 5 ms (2-1-2)	50/s	110- 0 dB peSPL (in 10 dB)	10, 12, 16, 20, 24, and 32 kHz	Data not available	1000	HP-300 Hz, LP-3000 Hz (data not available)	3: head-permanent electrodes (reference and active), hind paw (ground)	Data not available
van Zwieten et al. 2019 [51]	Data not available	directly to ear canal	tone burst/ 5 ms (2-1-2)	50/s	110- 0 dB peSPL (in 10 dB)	10, 12, 16, 20, 24, and 32 kHz	Data not available	1000	HP-300 Hz, LP-3000 Hz (data not available)	3: head-permanent electrodes (reference and active), hind paw (ground)	Data not available
Ahsan et al. 2018 [49]	TDT	Data not available	click and tone burst/ 10 ms (0.5-9-0.5)	Data not available	100-5 dB peSPL (in 5 dB)	4, 8, 10, 16, 20, 24 and 30 kHz	Data not available	300-400	HP-300 Hz, LP-3000 Hz (60 Hz)	3: vertex (active), pinnas (reference and ground)	Data not available
Turner and Larsen, 2016 [65]	TDT	directly to ear canal	tone burts/ 5 ms (2-1-2)	29/s	95-5 dB SPL (in 10 dB)	8, 10, 12, 16, 20, 24 and 32 kHz	Data not available	512	HP-100 Hz, LP-3000 Hz (data not available)	3: around the first cervical vertebra, the dorsal cranial midline, rear leg (ground)	Data not available
Bing et al. 2015 [33]	Data not available	free field (data not available)	click, noise (1 ms) and tone burst/ 3 ms (1-1-1)	Data not available	100-0 db SPL (in 5 dB)	1-50 kHz	Data not available	64-256	Data not available	3: vertex (active), mastoid (reference), back (ground)	Data not available
Zheng et al. 2015 [59]	Data not available	directly to ear canal	tone burst/ 5 ms (2-1-2)	50/s	100-20 (in 20, 10 and 5 dB)	8, 16, 20, and 32 kHz	Data not available	Data not available	Data not available	3: vertex (active), mastoid (reference), occiput (ground)	Data not available
Zheng, McPherson and Smith, 2014 [58]	Data not available	directly to ear canal	tone burst/ 5 ms (2-1-2)	21/s	90-0 db SPL (in 5 dB)	8,16,20 and 32 kHz	Data not available	Data not available	Data not available	3: vertex (active), mastoid (reference), occiput (reference)	Data not available
Laundrie and Sun, 2014 [38]	TDT	Data not available	tone burst/ 5 ms (1-3-1)	21/s	80-0 dB SPL (in 10 dB d)	6, 12, 16 and 20 kHz	Data not available	Data not available	HP-100 Hz, LP-3000 Hz (data not available)	surgically implanted chronic electrode (no details)	Data not available

Ropp et al. 2014 [84]	TDT	directly to ear canal	tone burst/ 5 ms (0.5-4-0.5)	30/s	60- -20 dB SPL (Data not available)	2.5-40 kHz	Data not available	300	HP-300 Hz, LP-3000 Hz (data not available)	3: vertex (active), mastoid (reference), ipsilateral leg (ground)	maintaining body temperature at 37°C (heating pad)
Ruttiger et al. 2013 [60]	Data not available	free field: 3 cm lateral to the animal's pinna	click and tone burst/ 5 ms (1-3-1)	10/s or 80/s	click: 0–90 dB SPL, tone burst: 20–100 dB SPL (in 5 dB)	1-45 kHz	alternating	64-256	HP-200 Hz, LP-5000 Hz (data not available)	3: vertex (active), tested ears (reference) contralateral pina (ground)	Data not available
Pace and Zhang, 2013 [64]	TDT	directly to ear canal	click and tone burst/ 10 ms (0.5-9-0.5)	50/s	80-5 dB peak SPL (in 5 dB)	8, 12, 16 and 28 kHz	Data not available	300-400	HP-300 Hz, LP-3000 Hz (60 Hz)	3: vertex (active), ipsi and contr-pinnas (reference and ground)	Data not available
Singer et al. 2013 [71]	Data not available	open field: 3 cm lateral to the animal's pinna	click	10/s	100-0 dB SPL (in 5 dB)	< 4 kHz	alternating	64-256	HP-200 Hz, LP-5000 Hz (data not available)	3: vertex (active), ear (reference), back (ground)	Data not available
Brozoski et al. 2012 [63]	TDT or IHS	open field: 10 cm in front of the animal's head	click and tone burst/ 10 ms (0.5-9-0.5)	Data not available	90-0 db SPL (in 5 dB)	8, 10, 12, 16, 20, 24 and 32 kHz	Data not available	512	HP-100 Hz, LP-3000 Hz (Data not available)	3: vertex (active), mastoid (reference), left front paw (ground)	Data not available
Zheng et al. 2012 [70]	Data not available	directly to ear canal	tone burst/ 5 ms (2-1-2)	50/s	80-10 db SPL (in 5 dB)	8, 16 and 20 kHz	Data not available	Data not available	Data not available	3: vertex (active), mastoid (reference), back (ground)	Data not available
Zheng et al. 2012 [57]	Data not available	Data not available	tone burst/ 5 ms (2-1-2)	50/s	90-0 db SPL (in 5 dB)	8,16 and 20 kHz	Data not available	Data not available	Data not available	3: vertex (active), mastoid (reference), occiput (ground)	Data not available
Zheng et al. 2011 [56]	Data not available	directly to ear canal	tone burst/ 5 ms (2-1-2)	50/s	80-10 dB SPL (in 5 dB)	8, 16 and 20 kHz	Data not available	Data not available	Data not available	3: vertex (active), mastoid (reference), occiput (ground)	Data not available
Wang et al. 2009 [66]	IHS	Data not available	click and tone burst/ 5ms (2.5-0-2.5)	50/s	80-0 dB SPL (in 10 dB)	4, 10, 16, 20, 24 and 32 kHz	Data not available	1024	HP-100 Hz, LP-3000 Hz (data not available)	3: vertex (active), mastoid (reference), hind leg (ground)	Data not available

Ouyang 2017 (39)	TDT	directly to ear canal	click and tone burst/ (data not available)	Data not available	100- 5 dB SPL (in 5 dB)	8, 12, 16, 20 and 28 kHz	Data not available	300-400	HP-300 Hz, LP-3000 Hz (60 Hz)	3: vertex (active), pinna (reference) contr- temporal muscle (ground)	warm blanket to sustain the body temperature
Mahmood et al. 2014 [40]	TDT	directly to ear canal	click and tone burst/ 10 ms (data not available)	Data not available	90 -10 dB SPL (data not available)	8,12,16,20 and 28 kHz	Data not available	300-400	HP-300 Hz, LP-3000 Hz (60 Hz)	3: vertex (active), ipsi- and contr- pinnas (reference and ground)	warming blanket connected to a thermostatic controller to maintain body temperature
Mao et al. 2012 [41]	TDT	Data not available	click	50/s	80-5 dB peSPL (in 5 dB)	<4 kHz	Data not available	300	HP-300 Hz, LP-3000 Hz (60 Hz)	3: vertex (active), pinna (reference) contr- temporaris muscle (ground)	a warming blanket connected to a homeothermic control unit to maintain body temperature

Abbreviations: LP, low pass filter; HP, high pass filter; TDT, Tucker-Davis Technologies; HIS, I*ntelligent Hearing Systems*; ipsi, ipsilateral; contr, contralateral.
